# Direct Binding of a Hepatitis C Virus Inhibitor to the Viral Capsid Protein

**DOI:** 10.1371/journal.pone.0032207

**Published:** 2012-02-28

**Authors:** Smitha Kota, Virginia Takahashi, Feng Ni, John K. Snyder, A. Donny Strosberg

**Affiliations:** 1 Department of Infectology, The Scripps Research Institute, Scripps-Florida, Jupiter, Florida, United States of America; 2 University of Paris-Denis-Diderot-P7, CNRS and INSERM, Institut Cochin, Paris, France; 3 The Department of Chemistry, Boston University, Boston, Massachusetts, United States of America; The Scripps Research Institute, United States of America

## Abstract

Over 130 million people are infected chronically with hepatitis C virus (HCV), which, together with HBV, is the leading cause of liver disease. Novel small molecule inhibitors of Hepatitis C virus (HCV) are needed to complement or replace current treatments based on pegylated interferon and ribavirin, which are only partially successful and plagued with side-effects. Assembly of the virion is initiated by the oligomerization of core, the capsid protein, followed by the interaction with NS5A and other HCV proteins. By screening for inhibitors of core dimerization, we previously discovered peptides and drug-like compounds that disrupt interactions between core and other HCV proteins, NS3 and NS5A, and block HCV production. Here we report that a biotinylated derivative of SL209, a prototype small molecule inhibitor of core dimerization (IC_50_ of 2.80 µM) that inhibits HCV production with an EC_50_ of 3.20 µM, is capable of penetrating HCV-infected cells and tracking with core. Interaction between the inhibitors, core and other viral proteins was demonstrated by SL209–mediated affinity-isolation of HCV proteins from lysates of infected cells, or of the corresponding recombinant HCV proteins. SL209-like inhibitors of HCV core may form the basis of novel treatments of Hepatitis C in combination with other target-specific HCV drugs such as inhibitors of the NS3 protease, the NS5B polymerase, or the NS5A regulatory protein. More generally, our work supports the hypothesis that inhibitors of viral capsid formation might constitute a new class of potent antiviral agents, as was recently also shown for HIV capsid inhibitors.

## Introduction

Hepatitis C chronically infects over 130 million people worldwide [1]–[2]. There is no vaccine available and standard-of-care treatment is based on a combination of pegylated interferon and ribavirin, which has a poor response rate and is plagued with severe side-effects [3]–[4]. The search for targeted therapeutics for HCV has reached a major milestone with the recent FDA approval of two specific protease inhibitors [5]–[6], nearly ten years after the initial discovery of the efficacy of such agents [7]. Initially, these new drugs will still need to be administered in combination with the standard-of-care combination of pegylated interferon and ribavirin. The next advance will most likely be the replacement of the non-selective interferon by a second targeted antiviral, directed against another HCV protein, the RNA-dependent RNA polymerase, NS5B [8]–[10] and if necessary, a third antiviral, eg. the most recent discovered inhibitor of the regulatory protein NS5A [11]–[12].

A number of obstacles remain. The new anti-NS3 protease drugs are selective for genotype 1, where the greatest need exists in the Western countries, since more than half of patients infected with strains of this genotype are not cured by the interferon plus ribavirin combination. Even though genotype 1 infections constitute more than half of all cases, there are five other major HCV genotypes for which novel pan-genotypic drugs are urgently needed. Furthermore, the use of target-specific treatments inevitably leads to emergence of resistant strains, and the first mutants have already been reported [13]–[14]. Therefore it will be necessary to continuously develop novel combination therapies involving drugs directed against multiple targets.

Core, the capsid protein of HCV, could be a valuable target for such future drug development [15]. Core is responsible for assembly and packaging of the HCV RNA genome to form the viral nucleocapsid [16]. Core dimers and higher-order oligomers associate on lipid droplets and endoplasmic reticulum with other HCV proteins thus acting as essential elements of viral particle assembly possibly through dimerization-driven interaction with NS3 [17] and other HCV proteins, including NS5A [18]. Core is the least variable of all ten HCV proteins in clinical isolates of infected patients, and is very well conserved among the six HCV genotypes. Core plays a key role in the HCV life cycle during assembly and release of the infectious particle [19]. Inhibitors of capsid assembly may interfere with both uncoating of the viral particle upon infection, formation of new particles and even destabilization of assembled virions, as was recently demonstrated for an inhibitor of HIV capsid dimerization ([20]; Kota and Strosberg, unpublished results).

Inhibition of HCV core dimerization by peptides was reported previously [21]. Transfer-of-energy assays revealed that the N-terminal 106 residue fragment of core (core106) is sufficient to achieve 91% inhibition, and that 15- to 18-residue peptides derived from the homotypic region (positions 82–106) inhibited respectively 50 to 68% of core dimerization (IC_50_ of 20.9 µM) [21]–[22]. Physicochemical properties of binding of the peptides to core were measured by Fluorescence Polarization Light analysis (apparent Kd of 1.9 µM), and by Surface Plasmon Resonance characterization of binding to mature core (apparent Kd of 7.2 µM [21]). Drug-like small molecules, identified using the assays developed to characterize the core-derived peptide inhibitors, displayed half-maximal inhibition of core dimerization and HCV infectivity at 90 nM concentrations [23]. However, evidence for direct binding to HCV core protein in cells has lacked so far. We show here that a biotinylated derivative of SL209, one of these small molecule inhibitors, directly binds to HCV core presumably at the site of viral assembly in infected cells. Ligand-based affinity isolation performed on lysates of HCV-infected cells or on recombinant HCV proteins demonstrated that the presence of core is required to retain other HCV proteins on the affinity-gel, thus confirming the central role of core in virion assembly.

## Materials and Methods

### Compounds, Proteins, Antibodies, Cells, Replicon, and Viruses

Compounds SL201, SL209 and SL231, and analogues were made at the Center for **C**hemical **M**ethodology and **L**ibrary **D**evelopment (CMLD) at Boston University (BU), Boston, and their synthesis was described previously as compound 15, and 17 in Wei et al., 2009 and as compound 1 and 2 in Ni et al., 2011 [23]–[24] respectively. SL209-biotin was prepared as indicated below. HCV core106 (1–106 residues) [21] and core169 (1–169 residues) [21], NS3 helicase (167–631 residues) [17] and NS5A (30–447 residues) [25], as well as their GST and Flag-tagged versions of proteins were produced in E. coli and purified by Ni-NTA affinity chromatography as described previously [17], [21]–[22], [25]. NS5A protein was provided by Drs. I. Herrera-Angulo and T. Tellinghuisen (TSRI-Florida). Antibodies were obtained from commercial sources: anti-GST (Sigma–Aldrich), anti-core (MA1-080) (Thermo Scientific), anti-NS3 [H23] (Ab138830-Abcam), anti-LAMP1 (ab-24170-Abcam), horseradish peroxidase (HRP)-conjugated secondary antibodies (Jackson ImmunoResearch) or as a gift: anti-core polyclonal (R-308) from Dr. J. McLauchlan, anti-NS5A [9E10] from Dr. T. Tellinghuisen. The hepatoma Huh-7.5 cells were kindly provided by Dr. F. Chisari, (TSRI, California). The HCV 2a genotype pSGR-JFH1 subgenomic replicon, the HCV 2a strain JC1 were kindly provided by Dr. T. Tellinghuisen, Dr. C. Rice and Dr. T. Wakita.

### Synthesis of Biotinylated SL209

*Dimethyl8-(5-aminopentyl)-4-benzyl-3-oxo-1,2,3,4,7a,8-hexahydroindo-lo[2,3-d][1,8]naphthyridine-6,7-dicarboxylate (* ***2*** *)*. The mixture of azide **1** (Ni et al, 2011 [23], 37.0 mg, 66 µmol), triphenylphosphine (52.3 mg, 0.20 mmol) and water (12.0 mg, 0.66 mmol) in THF (1 mL) was allowed to stir at 50°C for 5 hr in a sealed microwave tube. The solution was then concentrated under high vacuum to remove the solvent, and the residue was purified via flash chromatography on silica gel, eluting with dichloromethane∶methanol (5∶1, Rf 0.1) to give crude amino compound as a yellow oil (35.0 mg), which was used in the next step without further purification.

**Figure 1:**
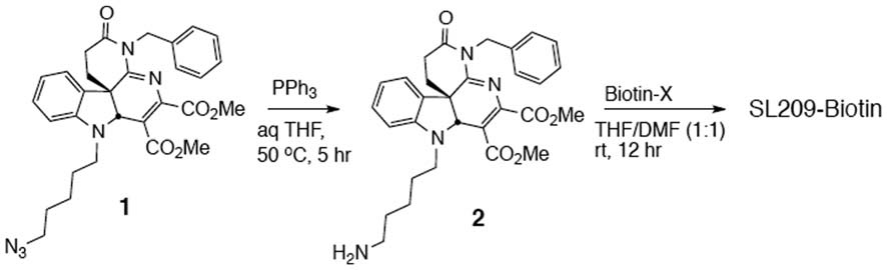
Scheme for synthesis of Biotinylated SL209.

*Biotinylated SL209*. A mixture of amine **2** (35.0 mg, 66 µmol) and Biotin-X (30.0 mg, 66 µmol) in anhydrous THF/DMF (1∶1, 1 mL) was allowed to stir at room temperature overnight (12 hour) in a sealed microwave tube under argon. The solution was concentrated under high vacuum and the residue was purified *via* prep: HPLC (acetonitrile in H_2_O 10–40%) to give biotinylated SL209 as a yellow oil (37.0 mg, 71% over 2 steps), SiO_2_ Rf = 0.4 (dichloromethane∶methanol = 5∶1). Purity (100%) confirmed by LC-MS (Fig. 1).

### AlphaScreen assay

The core106 **A**mplified **L**uminescent **P**roximity **H**omogenous **A**ssay screen (AlphaScreen) assay was developed as described in Kota et al 2009 [21]. AlphaScreen utilizes photoactive donor and acceptor beads which recognize specific tags on interacting proteins [26]. The biological interaction between proteins brings the beads in close proximity, and triggers a cascade of chemical reactions. When the donor bead is laser-excited at 680 nm, it converts the ambient oxygen into singlet oxygen which reacts with the chemiluminescer in the acceptor bead which in-turn activates fluorophores contained within the same acceptor bead to emit light at 520–620 nm. Singlet oxygen has a very short half life and cannot diffuse more than 200 nm. Hence this cascade of reactions can only occur when the interacting proteins are in close proximity. For the core106 AlphaScreen the recombinant purified proteins were diluted to working concentrations in ‘protein buffer’ (100 mM HEPES pH 7.5, 1 mM EDTA, 5 mM DTT, 0.1% CHAPS, 10% glycerol). The donor and acceptor beads were diluted to working concentrations in ‘bead buffer’ (20 mM HEPES pH7.5, 125 mM NaCl, 0.1% BSA, 0.1% CHAPS). GST-fused core106 and Flag-fused core106 were used at a concentration of 150 nM each. The untagged core106 domain (10 µM) was added to the proteins as a reference inhibitor, or SL209 (15 µM), or SL209-biotin (15 µM) were evaluated on core–core interaction. GST-core106, inhibitors and then Flag-core106 were incubated at room temperature for 1 hour. Then anti-Flag acceptor beads (Perkin Elmer Life Sciences), diluted to a final concentration of 20 µg/ml in bead buffer were added to the plate and incubated for 1 hour at room temperature. Finally, glutathione donor beads (Perkin Elmer Life Sciences) were added to the proteins at a final concentration of 20 µg/ml. The assays were executed in a white 384 well Packard opti plate (Perkin Elmer Lifesciences) and were read on Perkin Elmer Envision. The data of the uninhibited control compared to the inhibition by either SL209 or SL209-biotin were analyzed using unpaired student t-test. SL209 and SL209-biotin were also dosed with a 4-fold dilution from 160 µM to 150 pM. The IC_50_ values were calculated using GraphPad Prism.

### Co-precipitation

Huh-7.5 hepatoma cells were grown for 24 hours before electroporation with infectious HCV. At day 4 post-electroporation, the supernatant was removed and cells were lysed in 400 µl of lysis buffer (PBS with 1% Triton X-100, 1 mM PMSF, and protease inhibitor cocktail) and stored at −20°C. The presence of HCV proteins core, NS3 and NS5A in cell lysate was verified by immunoblotting using anti-core, anti-NS3 and anti-NS5A antibodies. For Streptavidin pull-down, 50 µM of SL209-biotin was immobilized on Streptavidin-agarose beads and was incubated with 80 µl of cell lysate in incubation buffer (20 mM Tris, pH8.0, 150 mM NaCl, 10% glycerol, 0.05% Triton X-100) for 2 hours at room temperature under end-over-end agitation. As control, 50 µM of free biotin was incubated with cell lysates. The beads were washed 3 times and centrifuge at 5000 g for 1 minute using a SigmaPrep™ spin column and boiled in the SDS loading buffer for 5 minutes, and the pulled-down proteins were detected by immunoblotting, using specific antibodies. One-twentieth of the lysates was included in the blots as input control. For the co-precipitation of core106 or core169 by SL209-biotin, 50 µM of SL209-biotin was incubated with 10 µg of core106, core169 or control protein CTD (C-Terminal Domain of HIV capsid protein CA). To investigate whether SL209-biotin binds NS3 and NS5A directly, 50 µM of SL209-biotin was immobilized on Streptavidin agarose beads and was incubated with either 80 µl of PSGR replicon lysates or 10 µg of either recombinant NS3 or NS5A. To confirm the need for core in mediating the binding of SL209-biotin to NS3 or NS5A, 10 µg of core106 was spiked into the replicon lysates and co-precipitation was carried out as described above for the replicon lysate. As a control, 10 µg of CTD was spiked into the replicon lysate and co-precipitated with SL209-biotin.

### Immunoblotting analysis

HCV lysate proteins were separated electrophoretically on a 4–20% Tris glycine gradient gel by SDS-PAGE, transferred onto a nitrocellulose membrane which was saturated with 5% milk in PBS at room temperature for 1 hour. After incubation with alternate protein-specific primary antibodies overnight, the membrane was washed three times with PBS containing 0.01% tween-20 and three times with PBS. Species-specific HRP-tagged secondary antibody was added to the membrane at room temperature for 2 hours. After the incubation, the membrane was washed three times with PBS containing 0.01% tween-20 and three times with PBS. Then the membrane was developed using a HRP-specific chemiluminescent substrate.

### Cell Culture and virus production

HCV was produced using previously published protocols [27]–[29] and [30]–[32]. The plasmid containing HCV RNA was linearized using XbaI and the overhangs were removed by addition of mung bean nuclease. The templates were purified using a PCR cleanup kit (Qiagen, Valencia,CA) and 2 µg of this reaction was used as a template for RNA transcription. The transcription reaction was performed using a T7 RNA transcription kit (Ambion, Ausitn,TX). After a 3 hour synthesis reaction at 37°C RNA was DNase-treated and purified using Rneasy minicolumns (Qiagen, Valencia, CA). RNA concentrations were analyzed by specotrophotometry and stored frozen at −80°C until use. The Huh-7.5 hepatoma cells were routinely cultured in Dulbecco's modified Eagle medium supplemented with 10% heat inactivated fetal bovine serum, 1X Pen-Strep-Gln and 1X non-essential amino acid at 37°C with 5% CO_2_ and 95% relative humidity in 15 cm dishes. For RNA transfection, cells were washed with phosphate buffered saline (PBS), trypsinized, and resuspended in complete growth medium. Cells were then pelleted, washed with ice-cold PBS, and resuspended in ice-cold PBS at 5×10^6^ cells/ml. 10 µg of RNA transcripts were mixed with 400 µl of cells and placed into a 2 mm gap electroporation cuvette (BTX Genetronics, San Diego, CA) and electroporated with five pulses of 99 µSec at 860 V over 1.1 seconds in an ECM830 electroporator (BTX Genetronics). After a 30 minute recovery period, cells were mixed with complete growth medium and plated. Subgenomic replicon pSGR-JFH1 cell line was grown under 1 mg/mL G418 selection in the same conditions as the Huh-7.5 cells.

### Immunofluorescence Microscopy

Naïve Huh-7.5 cells were infected with HCV with or without SL209-Biotin in LabTek II 4-well chamber slides for 3 days. Infected cells were washed twice with PBS (phosphate-buffered saline) and then fixed with fixative solution, permeabilized with digitonin, and blocked following the protocol previously published [33]. After washing with PBS, cells were incubated with anti-core (MA1-080 or R308) or anti-NS5A (9E10) primary monoclonal antibodies and then incubated with anti-mouse secondary fluorescent antibodies Alexa555 and streptavidin-Alexa488 (Invitrogen, Carlsbad, CA) for 1 hour at room temperature. Cells were finally washed with PBS and mounted with Prolong Gold mounting medium with DAPI (Invitrogen, Carlsbad, CA). Images were taken on an Olympus FV1000 confocal laser scanning microscope (Olympus Corporation, Tokyo, Japan). For visualization of the lipid droplets, cells were stained with BODIPY lipid probes 556/568 (Invitrogen, Carlsbad, CA). The percent inhibition of HCV infection by SL209 in IHC data was determined by microscopic observation.

### Inhibition of HCV production in Huh-7.5 cells

HCV infectious supernatant was made by electroporation of HCV RNA into naïve Huh-7.5 cells in 15 cm dishes, incubated for 72 hours, and then infectious culture supernatants were collected and spun to remove cell debris. The viral titer was calculated by limiting dilution assay (see below) and all inhibition assays were performed at a Multiplicity Of Infection (MOI) of 5 to 8. For inhibition assays, naïve Huh-7.5 cells were seeded into 24-well plates and incubated for 24 hours to allow adhesion. The compound was prepared in HCV-infected cell supernatant by making 1∶10 serial dilutions from 100 µM down to 0.001 µM, was added to cells and incubated for 24 hours. The next day, cell culture media was removed from each well and replaced with the same dilutions of compound in fresh complete media, added to cells, and incubated for another 48 hours (previously referred to as T1) [17], [23]. Supernatant from this time point was then transferred to naïve cells for 24 hours before removing culture media and replacing with fresh complete media, without adding new compound for the remainder of 72 hours (previously referred to as T2) [17], [23]. Two stages of viral infection were studied: T1, corresponding to the initial 72 hour culture of naïve cells infected in the presence of the inhibitor, and T2, corresponding to the second 72 hour passage through naïve cells [17].

### Quantitative Real Time RT-PCR analysis of HCV RNA

Cells were lysed and RNA was isolated using the RNeasy kit (QIAGEN, Valencia, CA). DNA was generated using the Taqman reverse transcription kit (Applied Biosystems, Foster City, CA). Quantitative Real-Time polymerase chain reaction (PCR) was performed in triplicate using LightCycler RNA Amplification Kit HybProbe master mix (Roche) with Taqman MGB Probe 6FAM-TATGAGTGTCGTGCAGCCTC-MGBNFQ on a model LightCycler480 Real-Time PCR system (Roche). Data are expressed as the mean fold change plus or minus standard error of 3 replicates normalized to 100 µg total RNA. Primers used were forward CTTCACGCAGAAAGCGTCTA and reverse CAAGCACCCTATCAGGCAGT.

### Cytotoxicity Assay

To perform a XTT-based (2,3-bis(2-methoxy-4-nitro-5-sulfophenyl)5-[(phenylamino) carbonyl]-2H-tetrazolium hydride) cytotoxicity assay [34], 8,000 cells in 100 µL volume of colorless growth media were dispensed into each well of 96-well tissue culture-treated microtiter plates and incubated overnight at 37°C (5% CO2, 95% RH) to allow cells to adhere to the plate. The next day, 100 µL of compound SL209 in DMSO (1.25% final DMSO concentration) or DMSO alone were added to wells. Growth medium with compound only was used to normalize the data to account for background color. Next, the plates were incubated for 72 hours at 37°C (5% CO2, 95% RH). Solution of XTT-PMS (phenazine methosulphate) was prepared in 1X PBS and added to the cells in 50 µL volume to each well and incubated for 4 hours at 37°C (5% CO2, 95% RH). After equilibrating the plates to room temperature for 10 minutes, optical density at 450 nm and 650 nm was measured on Biotek plate reader.

### HCVcc Limiting dilution assay (Tissue Culture Inhibition Dose-TCID_50_)

After wells were coated with poly-L-lysine (Sigma), naïve Huh-7.5 cells were seeded at 8000 cells per well in 96-well plates and incubated overnight at 37°C. Supernatants collected from HCV-infected Huh-7.5 cells in presence or absence of compound were titrated with serial 1∶10 dilutions, from 0 to 10-6 dilution, in complete media and added onto Huh-7.5 cells for 72 hours in the HCVcc limiting dilution assay to determine TCID_50_ values. After 72 hours, cell supernatant was removed from each well and cells were fixed with 100% cold methanol for 10–20 minutes at room temperature. Immunohistochemistry was then performed on fixed cells beginning with washing cells three times with PBS, then followed by blocking for 1 hour at room temperature with blocking buffer containing 0.5% (w/v) saponin, 1% (w/v) bovine serum albumin (BSA), and 0.2% (w/v) dried non-fat milk in PBS. Cells were incubated with 0.3% (v/v) hydrogen peroxide for 5 minutes at room temperature to block endogenous peroxidases before washing cells three times with PBS. Cells were then incubated with mouse 9E10 anti-NS5A antibody at a dilution of 1∶20,000 in PBS buffer containing 0.5% (w/v) saponin for 1 hour at room temperature or overnight at 4°C and washed three times with PBS. Secondary goat anti-mouse conjugated with horse horse radish peroxidase (HRP) antibody was prepared at 1∶200 dilution in PBS buffer containing 0.5% (w/v) saponin and incubated on cells for 1 hour at room temperature. Cells were washed three times in PBS. Bound peroxidase was developed with DAB chromagen containing diaminobenzidine tetrahydrochloride (Vector Laboratories, Burlingame, CA). Wells with at least one positive staining of NS5A were counted and the method of Reed and Muench was used to calculate TCID50 values. Cell nuclei were stained with hematoxylin 2 to record images (Richard Allan Scientific, Kalamazoo, MI) under microscope [32].

## Results

### Biotinylation of small molecule SL209 does not affect inhibition of dimerization

**Figure 2:**
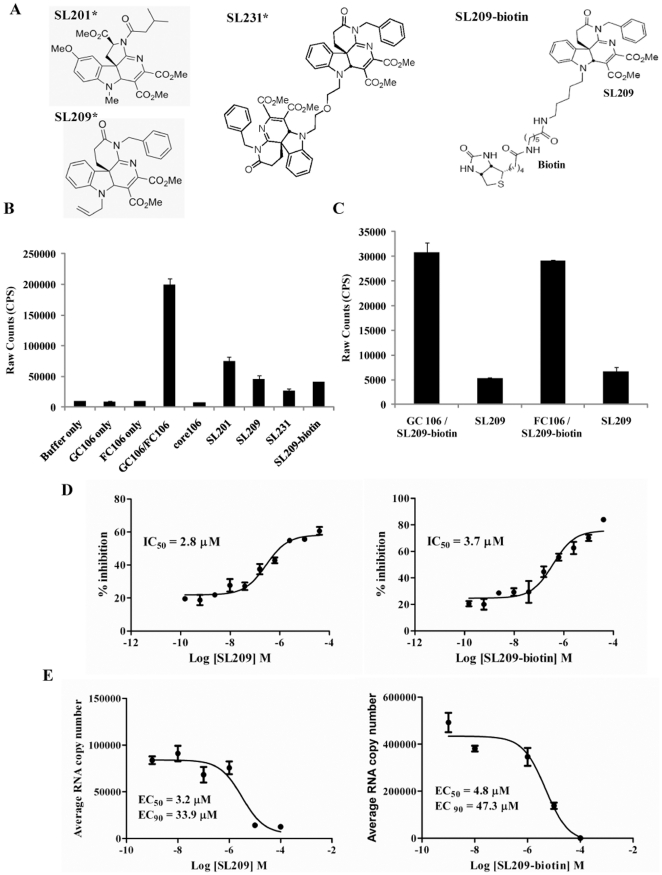
Inhibition of core dimerization and of virus production by SL209-biotin and evidence for direct binding to core protein. In an AlphaScreen assay, glutathione-coated donor beads and anti-flag antibody coated acceptor beads (20 µg/ml each) were used to monitor the dimerization of GST- and Flag- tagged core106 proteins. GST-core106 (GC106) and Flag-core106 (FC106) were used at 150 nM each and core106 was used at 1 µM as a reference inhibitor. **A: Structures of SL201, SL209, SL231, and SL209-biotin.** SL201, is a 513 Da small molecule inhibitor originally identified to inhibit HCV core dimerization and virus production. SL209, is a SAR analogue of SL201. SL231, is a dimer of SL209. SL209-biotin is a biotinlyated derivative of SL209. “*” indicates that structures of SL201, SL209, and SL231 have been previously published in Wei et al 2009 [24], Strosberg et al 2010 [15], and Ni et al 2011 [23]. **B: Levels of inhibition.** Core106 inhibited 91% and small compounds inhibitors: SL201, SL209, SL231 and SL209-biotin used at 15 µM inhibited respectively 66%, 74%, 83%, and 75% of core dimerization. **C: Direct binding to GST-core106 (GC106) or Flag-core106 (FC106).** In a novel AlphaScreen format GC106/SL209-biotin and FC106/SL209-biotin were mixed in 1∶1000 ratio and incubated. Streptavidin donor beads and glutathione coated acceptor beads at 20 µg/ml were used in the detection of the binding. Free SL209 at 50 µM inhibited 83% of SL209-biotin binding to GC106 and 77% of SL209-biotin binding to FC106. **D: Dose response analyses.** Inhibition levels were analyzed in a dose response format. The compounds were dosed from 160 µM to 150 pM. The IC_50_ values for SL209 (right panel) and SL209-biotin (left panel) were calculated as 3.7 µM and 2.8 µM using GraphPad Prism. **E: Inhibition of HCV production.** Inhibition of HCV production in Huh-7.5 cells by SL209 and SL209-biotin was analyzed by adding serially diluted the compounds (individually) and virus onto naïve Huh-7.5 cells. The supernatants of the cells after 3 days of culture were removed from the initial culture and added to naïve cells cultured for another 3 days. RNA was purified from lysed cells and analyzed by Real-Time RT-PCR. EC_50_ values were calculated to be 3.2 µM and 4.8 µM for SL209 (right panel) and SL209-biotin (left panel), respectively. EC_90_ values were calculated to be 33.9 µM and 47.3 µM for SL209 (right panel) and SL209-biotin (left panel), respectively.

The AlphaScreen and TR-FRET assays developed for identifying peptide disruptors of core106 or core169 dimerization were used to identify small molecules with similar properties and lower IC_50_, including SL201, its more potent analogue, SL209, and the dimer of SL209, SL231.Their structures are presented in Figure 2A [17], [21]–[24]. To prove that small molecule inhibitors of core dimerization directly bind to core, we prepared a biotinylated derivative of SL209 (SL209-biotin) (Fig. 2A). We verified by the core106 AlphaScreen assay that addition of the biotin tag did not alter the inhibitory activity: SL209-biotin disrupts core dimerization by 75% compared to 83% for untagged SL209, 74% for SL231, and 66% for SL201, another analogue (Fig. 2B). To eliminate the remote possibility that SL209-biotin binds to either GST or Flag rather than to core106 itself, we developed two novel AlphaScreen assays, one with GST-core106 and the other with Flag-core106. Untagged SL209, when added at 10- to 15-fold excess, inhibited the binding of SL209-biotin by respectively 83% and 77% (Fig. 2C).

When analyzed by dose response studies, the IC_50_ value of SL209-biotin for inhibition of core106 dimerization was calculated to be 3.7 µM (Fig. 2D-right panel), compared to 2.8 µM for SL209 (Fig. 2D-left panel).

### SL209 and SL209-biotin reduce HCV RNA production and HCV infectivity

The effects of SL209 and its biotinylated analogue on the Huh-7.5 host cells of HCV were comparable: SL209-biotin was only slightly more toxic to Huh-7.5 cells. Biotinylation did not affect the capacity to reduce HCV RNA production, measured by Real Time RT-PCR for SL209-biotin (EC_50_ = 4.80 µM; EC_90_ = 47.3 µM) compared to SL209 (EC_50_ = 3.20 µM; EC_90_ = 33.9 µM) (Fig. 2E and Table 1).

**Table 1:** Anti-viral activity of SL209 and associated compounds.

Compound	Core dimerization IC_50_ [µM]	Cytotoxicity CC_50_ [µM]	HCV inhibition EC_50_ by RT-PCR [µM]	HCV inhibition EC_90_ by RT-PCR [µM]
SL201*	9.30	>320	8.80	92.80
SL209*	2.80	>100	3.20	33.90
SL231*	0.10	>100	2.50	25.90
SL209-biotin	3.70	>40	4.80	47.30

The compounds were analyzed in the core dimerization AlphaScreen assay to determine the IC_50_ values, on naïve Huh-7.5 cells to calculate the CC_50_ values and finally on HCV infected (Multiplicity Of Infection – MOI – 5 to 8) Huh-7.5 cells to determine the EC_50_ values using Real Time RT-PCR.

“*”indicates previously published in Wei et al. [24], Strosberg et al. [15], Mousseau et al. [17], Ni et al. [23].

### Streptavidin-agarose beads coated with SL209-biotin bind HCV proteins

**Figure 3:**
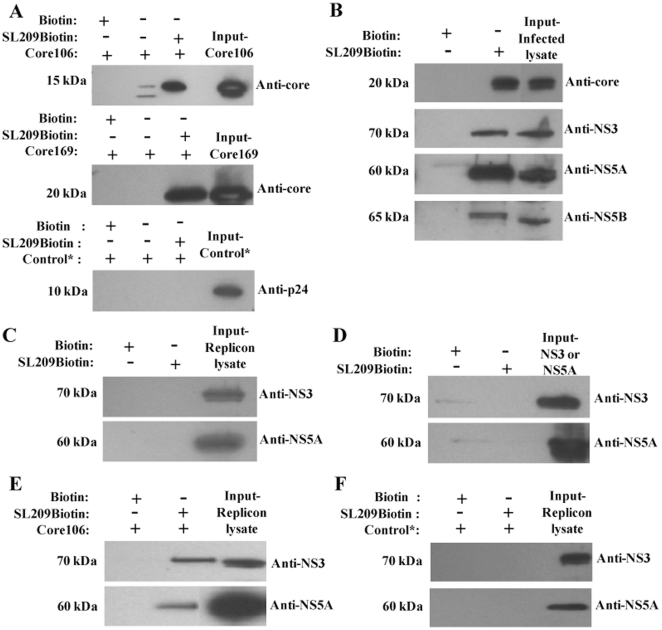
Affinity isolation on SL209-biotin captures native and recombinant HCV core and core-associated proteins. SL209-biotin was immobilized on streptavidin agarose beads and incubated with core106 (10 µg) or core169 (10 µg) proteins or lysates of Huh-7.5 cells infected with HCV J6/JFH-1. The retained proteins were examined by immuno-blotting using anti-core, NS3, NS5A or NS5B antibodies. One-twentieth of the cell lysate used in the assay was immuno-blotted for all proteins as the input control. As a control, the lysates were incubated with Biotin immobilized on streptavidin agarose beads. As an additional control, a non-specific protein, CTD (C-terminal domain of HIV capsid protein, p24) was incubated with immobilized SL209-biotin. **A:** SL209-biotin captures/co-precipitates recombinant core106 (top panel) and core169 proteins (middle panel) and does not capture non-specific control protein CTD (bottom panel). **B:** SL209-biotin captures/co-precipitates core, NS3, NS5A, and NS5B from HCV-infected Huh-7.5 cells, but **C:** not from replicon containing Huh-7.5 cell lysates. **D:** SL209-biotin does not co-precipitate recombinant NS3 and NS5A proteins. **E:** Core protein added to replicon-containing Huh-7.5 cell lysates rescues co-precipitation of NS3 and NS5A. **F:** CTD protein added to replicon-containing Huh-7.5 cell lysates does not rescue the co-precipitation of NS3 and NS5A.

SL209-biotin bound to streptavidin-covered agarose beads retains a 15 kDa band corresponding to recombinant core106 (Fig. 3A-top panel) and a 20 kDa band corresponding to core169 protein (Fig. 3A-middle panel), as shown by SDS-electrophoretic analysis followed by immunoblotting with anti-core antibodies. The biotin-only control did not retain either one of the proteins. The control protein HIV CTD (C-terminal domain of HIV core protein p24) was not retained by either biotin or SL209-biotin confirming the specificity of the binding of SL209-biotin to core (Fig. 3A-bottom panel). SL209-biotin retains native HCV NS3, NS5A, and NS5B proteins contained within HCV-infected cell lysates, in line with previously reported co-localization studies (Fig. 3B). The NS5A protein detected here by Western blotting is the hyper-phosphorylated form which runs at ∼58 kDa. The biotin-only coated streptavidin agarose beads did not retain any of the proteins contained within the HCV-infected cell lysates. No protein was captured on beads incubated with lysates of cells expressing HCV pSGR subgenomic replicon, devoid of core (Fig. 3C). SL209-biotin alone did not retain either NS3 helicase or NS5A confirming the need for the presence of core to establish the interaction (Fig. 3D). As further proof that core is required for binding of SL209 to NS3 and NS5A, the pSGR replicon cell lysate was spiked with recombinant core106, and analyzed by immunoblot: the capsid protein rescued the capture of NS3 and NS5A by SL209-biotin (Fig. 3E). As a non-related protein control, HIV CTD was spiked into the pSGR replicon lysate. Co-precipitation analysis was carried out with SL209-biotin immobilized on Streptavidin beads and incubated with replicon lysates spiked with CTD and the resulting eluates were analyzed by immunoblotting. CTD did not rescue the capture of NS3 and NS5A by SL209-biotin which confirmed that core is necessary for this interaction (Fig. 3F).

### Treatment with core dimerization inhibitors strongly reduces the number of HCV infected cells, visualized by anti-NS5A antibody

**Figure 4:**
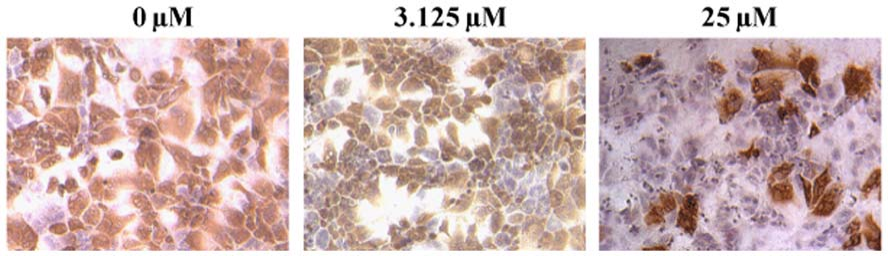
SL209 strongly reduces number of HCV-infected Huh-7.5 cells. Huh-7.5 cells seeded into 96-well plate were infected with HCV in the presence of increasing amounts of SL209 and incubated for 72 hours. Cells were fixed with methanol and immunohistochemistry was performed to stain cells for expression of the HCV NS5A protein with anti-NS5A (9E10) antibodies followed by rabbit anti-mouse HRP-conjugated secondary antibody. We show here three representative fields containing large number of infected cells of which treatment with SL209 progressively reduces numbers of cells stained with the anti-NS5A antibody. The left field (“no compound”) was treated with vehicle, and the following two fields were treated with 3.125 µM and 25 µM SL209. Wells treated with 25 µM of SL209 showed a substantial reduction in HCV-infected cells. The cells were counter stained with hematoxylin 2 to visualize the nuclei. Magnification is 10×. The data are representative of 4 wells in each experiment and 3 different experiments.

Huh-7.5 cells infected with HCV in presence of 0, 3, or 25 µM of SL209, were washed after 24-hours, supernatant was replaced with compound in fresh complete media and cells incubated for an additional 48 hours. The cytotoxicity index CC_50_ value for SL209 measured on uninfected cells was found to be above 100 µM and the EC_50_ value evaluated by Real Time RT-PCR on HCV-infected cells was found to be 3.2 µM [23]–[24]. Immunohistochemical staining of HCV NS5A in infected cells revealed that SL209 used at 3.125 and 25 µM reduced the number of HCV-infected cells substantially (Fig. 4). The results shown are representative of 3 experiments with 4 wells per experiment. The inhibition of HCV infection by SL209 in immunohistochemistry data was studied by microscopic observation. The EC_50_ value by Tissue Culture Infectious Dose _50_ limiting dilution assay was calculated to be 5 µM.

### Expression of core is required for intracellular staining by SL209-biotin combined with streptavidin-fluorochrome

**Figure 5:**
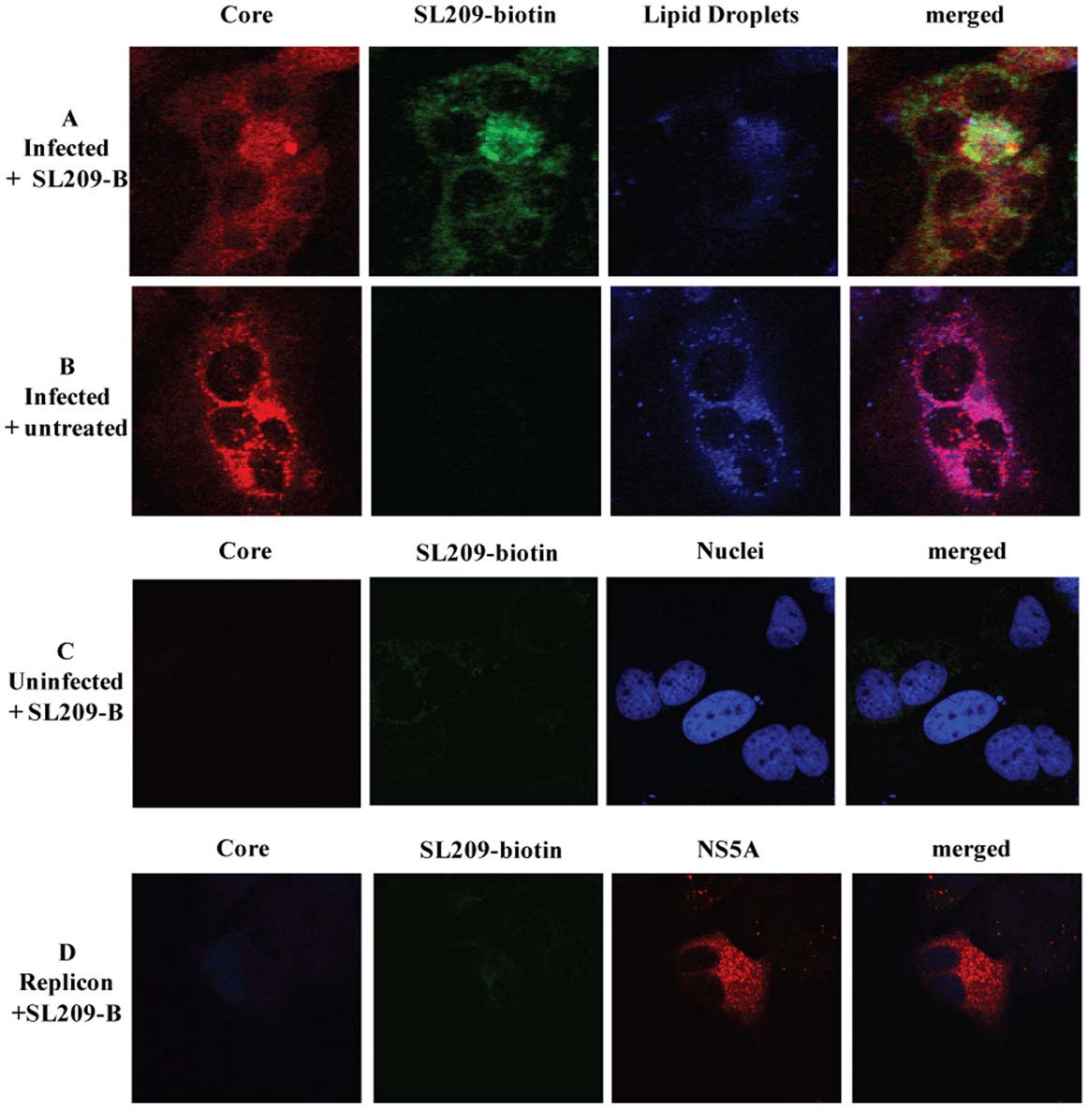
Core is required for labeling by SL209-biotin. Huh-7.5 cells seeded into chamber slides were infected with HCV in the presence of increasing amounts of SL209-biotin and incubated for 72 hours. Infected cells were probed for expression of core with anti-core antibody and replicon cells were probed for both core and HCV NS5A protein respectively with anti-core (R-308) anti-NS5A (9E10) antibodies. Alexa555-coupled secondary antibody was used to detect bound specific antibody. Huh-7.5 cells are stained with DAPI to localize nuclei, and studied by confocal microscopy. In immunofluorescence studies of HCV-infected cells, treated with 2 µM of SL209-biotin (below the EC_50_ value of 4.8 µM) and streptavidin-Alexa488 and labeled for core, SL209-biotin can be seen to penetrate the infected cells (**Fig. 5A**). No such labeling could be observed when compound is not present **(Fig. 5B)**, in uninfected cells (**Fig. 5C**), or in cells expressing HCV subgenomic replicon which produce NS5A but not core (**Fig. 5D**). Magnification was 10× with 3× optical zoom and the images were cropped to display the field of interest.

HCV-infected cells were treated with SL209-biotin and Streptavidin-Alexa 488, and then stained by anti-core fluorescent antibodies. The resulting characteristically dot-like images confirmed that the inhibitor was mostly found to be co-localized with core often but not always on lipid droplets (Fig. 5A). No streptavidin-mediated labeling was observed in untreated cells (no SL209-biotin, therefore no Streptavidin-Alexa488 staining) (Fig. 5B), in uninfected cells (Fig. 5C), or in cells expressing HCV subgenomic replicon (no core therefore no SL209-biotin binding, nor Streptavidin Alexa488 staining (Fig. 5D).

## Discussion

### Direct binding of inhibitors to core

We describe here the first evidence of binding, to the HCV capsid protein, of a core dimerization inhibitor which reduces HCV production and infectivity [23]. Direct binding was shown by using a biotinylated derivative of small molecule drug-like SL209, that largely maintained the HCV inhibitory properties of the untagged compound. Using SL209-biotin absorbed on agarose beads coated with streptavidin, direct physical interaction was demonstrated by affinity-isolation performed on lysates of HCV-infected cells, and confirmed with recombinant HCV proteins. Affinity-binding was shown not only for core, but also for other HCV proteins which were previously reported to be co-localized with core on lipid droplets or on ER, namely NS3, NS5A, and NS5B [18], [33], [35]–[36]. In the absence of core, neither NS5A nor NS3 were retained on the SL209-biotin coated streptavidin beads. These results confirm that in the affinity-isolation conditions, SL209-biotin binding is strong enough to retain core complexes containing other HCV proteins. In support of this observation, co-localization of core and NS5A proteins with SL209-biotin was occasionally observed in HCV-infected cells using confocal microscopy. Despite similar structures, SL209-biotin may thus differ significantly from another inhibitor SL201, which had earlier been shown to not only disrupt core dimerization, but also inhibit interaction with NS3 helicase [17]. Alternatively, assay conditions and their effect on avidity and affinity may play a major role in dictating complex formation: in cells, or on agarose beads. Protein-ligand complexes may be more stable than in the transfer-of-energy assays optimized to measure inhibition of protein-protein interactions [21]–[22].

### Site of binding of inhibitors on core

Our earlier data suggested that the core-derived peptide inhibitors bound to the core homotypic region, defined previously as being located between positions 82 and 106 [21], [37]. These peptides share the sequence LYGNEGCGWAGWLLSPRG, which was independently confirmed as an inhibitor of HCV RNA production and infectivity [38]. Our small compound inhibitors were identified using the same screening assays as the ones developed to identify the peptides [23]–[24]. However, these chemicals displayed IC_50_ values which were at least five times lower than those of the peptide, suggesting that binding occurs with higher affinity and possibly not on the same residues than the peptide. EC_50_ values for HCV inhibition were about three times and often much lower (nanomolar range) [23]. This could be attributed to the fact that the inhibitors disrupt core dimerization, a step which is necessary for core oligomers to form and remain stable. As stated previously for the HIV-1 CA-1 inhibitor, a very limited number of molecules may be sufficient to inhibit the formation of a functional particle, an observation consistent with the view that the whole viral capsid constitutes a single target with multiple binding sites [39].

### Intracellular labeling with biotinylated SL209 of HCV-infected cells

Using SL209-biotin in combination with Streptavidin-Alexa 488, we showed by confocal immunofluorescence microscopy that the presence of core is indispensable to observe any intracellular staining: no labeling is observed in uninfected cells nor in cells infected with HCV replicon or with HIV. Furthermore, SL209-biotin staining coincides mostly, but not always, with immunostaining of core. Cellular co-staining of core and SL209-biotin occurs often in sites where we could not detect lipid droplets, mainly on Endoplasmic Reticulum (ER), as determined by staining with LAMP1 (Lysosomal-associated membrane protein1), an ER marker protein (Takahashi and Strosberg–unpublished data). This observation is in line with the recent discovery that the core protein of high-titer JC1 recombinant HCV virus, used in our studies frequently exhibits an ER localization [40], rather than the predominant lipid droplet localization of the core protein of JFH1 virus described in previous co-localization studies in HCV-infected cells [33], [41] and core-transfected uninfected cells [42]. Our choice of using JC1 in preference to JFH1 was dictated by a much improved level of viral production: whether this explains a possible shift of virion production site and lower coating of lipid droplets is not known [40].

The work presented here supports core, the HCV capsid protein as a novel target for anti-HCV drug development. We show that an inhibitor of capsid protein dimerization can specifically and directly bind to core and core-based complexes with other HCV proteins. This binding possibly results in disruption of assembly or in disassembly of the viral particle, leading to reduction of infective HCV particles. One added advantage of HCV core over the other currently identified targets is its remarkable conservation among all six genotypes, especially in the previously described “homotypic” region of dimerization [37]. Inhibitors optimized on the basis of analogues described here have been found to be equally active on core proteins of genotype 1a or 1b and to inhibit virus production of a HCV 2a strain at nanomolar concentration. Despite several attempts, no resistance mutant were so far found to emerge rapidly in HCV 2a-infected cells grown in the presence of increasing concentrations of core inhibitors.

### Mechanism of action of SL209

While compound SL209 was identified as an inhibitor of core protein dimerization, the mechanism by which it blocks HCV production and infectivity is not yet known. Core multimerizes to form the capsid of the virus, which together with the viral RNA, forms the nucleo-capsid. Despite many attempts, core or even its N-terminal mostly hydrophilic fragment core106, could not be crystallized, so we do not yet have a good model for the assembled capsid. While we can exclude any effect SL209 has on entry (no effect on time-of-addition) [17] or replication (no effect on replicon propagation [17]), it is likely that the inhibitor of core dimerization acts on the viral capsid assembly or disassembly. By analogy with HIV capsid inhibitors [20], [39], [43], several alternative mechanisms can be considered for the molecular mode of action of SL209: inhibition of uncoating of the particle upon penetration, blockade of correct assembly of the capsid or destabilization of the assembled capsid.

### Capsid proteins as targets for viral inhibitors

Several groups have recently proposed viral capsid protein as targets for antiviral drug development for HBV and HIV. While capsid-derived natural or stapled peptides displayed relatively modest binding affinities, small compound inhibitors were described with quite impressive affinities, IC_50_ values in solution, and EC_50_ values in infected cells [39], [43]–[46]. Cell-based screening yielded small molecule compound PF-74, a potent inhibitor of HIV capsid assembly which was shown to have both early stage and late stage effects, in contrast to other compounds which only displayed late stage activity [20]. Co-crystallization of compound PF-74 with the HIV CA protein revealed a novel binding pocket distinct from the one identified earlier for peptides and *in silico* screened inhibitors [39], [43], [45]–[46]. The present work demonstrates a direct localization of a biotinylated derivative of a HCV inhibitor at the presumed site of viral particle assembly strongly supports the validity of capsid inhibitors as useful molecular probes to study capsid assembly and to serve as a basis for the development of potential new antiviral drugs.
